# Fathers’ sensitive parenting enhanced by prenatal video-feedback: a randomized controlled trial using ultrasound imaging

**DOI:** 10.1038/s41390-022-02183-9

**Published:** 2022-07-29

**Authors:** Renate S. M. Buisman, Kim Alyousefi-van Dijk, Noor de Waal, Ashwina R. Kesarlal, Martine W. F. T. Verhees, Marinus H. van IJzendoorn, Marian J. Bakermans-Kranenburg

**Affiliations:** 1 Clinical Child & Family Studies, Faculty of Behavioral and Movement Sciences, Vrije Universiteit, Amsterdam, The Netherlands; 2 Forensic Family and Youth Care Studies, Institute of Education and Child studies, Leiden University, Leiden, The Netherlands; 3 Anna Freud National Centre for Children and Families, London, UK; 4 Department of Psychology, Education and Child Studies, Erasmus University, Rotterdam, The Netherlands; 5 Research Department of Clinical, Education and Health Psychology, Faculty of Brain Sciences, UCL, London, UK; 6 Leiden Institute for Brain and Cognition, Leiden University, Leiden, The Netherlands; 7 Center for Attachment Research, The New School for Social Research, New York, USA

## Abstract

**Background:**

The aim of this study was to evaluate an interaction-based prenatal parenting intervention program aimed at promoting parental sensitivity and involvement in expectant fathers using ultrasound images: Prenatal Video-Feedback Intervention to Promote Positive Parenting (VIPP-PRE).

**Methods:**

In this randomized controlled trial, 73 first-time, healthy expectant fathers were enrolled. Participants were randomly assigned to the VIPP-PRE intervention (*n* = 39) or a dummy intervention (*n* = 34). Parental sensitivity was coded from video-recorded 10-min interactions with an infant simulator at a prenatal pretest and with fathers’ own infant at a postnatal posttest. Prenatal and postnatal involvement was assessed via an application on participants’ smartphones.

**Results:**

Fathers receiving VIPP-PRE demonstrated increased sensitivity across the perinatal period, relative to fathers receiving a dummy intervention. Fathers’ involvement with the infant increased significantly from the prenatal to postnatal period, regardless of the intervention.

**Conclusions:**

Prenatal video-feedback using ultrasound imaging of the unborn child has the potential to promote the quality of parenting in an important, but understudied, population and period: men in the transition to fatherhood. Future research should examine the long-term effectiveness of VIPP-PRE and its effectiveness in increasing parenting quality in at-risk families.

**Impact:**

- This study identifies a brief and focused prenatal intervention using assisted interactions between the father and his baby by means of ultrasound imaging as a promising strategy to improve sensitive fathering in the early postnatal phase.
- Our study shows that pregnancy provides a window of opportunity for promoting prenatal involvement and bonding in expectant fathers, with potential long-term benefits for the future father–child relationship.
- Ultrasound measures are currently used to monitor fetal growth and development, but our results suggest that they may also create an opportunity for stimulating father–infant interaction to promote postnatal caregiving quality.

## Introduction

Fathers, like mothers, are pillars in their children’s social and emotional development.^1^ Although fathers in several western countries have increased their participation in parenting over the past decades, with a three- to six-fold increase over one generation,^2^ their roles range widely from primary-caregiver fathers to absent fathers, and in most families, their parenting role is still modest.^3^ Also, research has repeatedly shown that fathers are less sensitive to their infants and toddlers than mothers.^4,5^ Thus, fathers’ involvement (representing the “quantity” of caregiving) and their parental sensitivity in the interaction with their children (representing the “quality” of caregiving) may benefit from intervention efforts specifically targeted at fathers. With the aim of supporting paternal involvement and sensitive caregiving from the earliest start of parenthood, we adapted the Video-Feedback Intervention to Promote Positive Parenting (VIPP^6^) for prenatal use (VIPP-PRE^7^) with fathers and tested its efficacy in a preregistered randomized controlled trial (https://osf.io/487xc).

Paternal sensitivity and engagement contribute substantially and independently from maternal sensitivity to children’s cognitive and socio-emotional development.^8–11^ Moreover, fathers’ sensitivity has been found to predict brain development both in the first year^12^ and in middle childhood.^13^ Thus, enhancing paternal sensitivity and involvement may benefit a broad range of child developmental outcomes.

Pregnancy provides a window of opportunity for promoting prenatal involvement and bonding in expectant fathers, with potential long-term benefits for the future father–child relationship^14^ and child developmental outcomes. Indeed, prenatal father involvement predicts postnatal father involvement^15^ and infant–father attachment.^16^ However, fathers are rarely involved in prenatal parenting interventions, and we know of only two parenting interventions that include fathers in the prenatal phase.^17,18^ Both are group interventions with educational sessions, aiming to increase paternal involvement in their children’s lives, but without opportunities for prenatal personal father–baby contact. Among parenting interventions, those using personalized video-feedback have been meta-analytically shown to be most effective in promoting parenting sensitivity.^19^ One of these programs, with documented effects on parental sensitivity (VIPP-SD, combined effect size based on twelve RCTs *d* = 0.47^20^) has shown its feasibility with fathers of toddlers.^21^ The switch to prenatal video-feedback, using ultrasound imaging of the child to facilitate parent-infant interaction is, to our knowledge, unique. Ultrasound imaging may help expectant fathers to develop an emotional bond with the unborn baby.^22^ This method of recording real-life interactions, providing protocolized but personalized feedback to make the parent aware of the dyadic nature of the interaction, and stimulating sensitive responses to the child’s behavior, has not yet been used to promote the father’s postnatal caregiving behavior. We hypothesized that fathers in the intervention arm, relative to fathers in the control arm, would show (1) a stronger increase in sensitivity from pretest to posttest, and (2) a stronger increase in involvement in caretaking activities from the prenatal to the early postnatal phase.

## Methods

### Participants

A total of 73 first-time expectant fathers participated in this study. The average years of education following primary school was 8.80 (SD = 1.44). Participants’ age ranged from 26 to 41 years (*M* = 32.62, SD = 3.26). At the time of inclusion, their partners were 18–31 weeks pregnant. At that time, we could confirm that their medical ultrasound showed no abnormalities (routinely done around 20 weeks), additionally, it would allow us the time needed to finalize pre-intervention and post-intervention measurements as well as the intervention before the baby was born. At pretest, the gestational age of their unborn infants, therefore, ranged from 20 to 32 weeks (*M* = 24.95, SD = 2.82). Partners of expectant fathers were low-risk women with uncomplicated singleton pregnancies, confirmed by a standard 20-week medical ultrasound. Participants received a travel allowance and a financial reward for their participation.

### Procedure

Recruitment took place via midwife agencies and other pregnancy-affiliated organizations. Eligible families received an invitation to participate with information about the study. Interested fathers received a letter and a phone call with more detailed information. Participants were included if they were (a) first-time expectant fathers, (b) sufficiently fluent in Dutch, (c) lived with their partner who had an uncomplicated pregnancy of a singleton and was between 18 and 31 weeks pregnant at the time of inclusion. Because of other measures that were part of this study fathers were excluded if they (a) had an endocrine or cardiovascular disease, (b) currently abused drugs or alcohol, (c) used medication potentially interfering with the endocrine system of neural activity, (d) had birth defects in the families of either parent, or (e) had partners using alcohol, tobacco, or illicit drugs during the pregnancy or with a BMI over 30 before pregnancy. The study procedure was registered at the Central Committee on Research Involving Human Subjects (CCMO, registry number NL62696.058.17) and approved by the Ethics Committees of the Leiden University Medical Centre and the Department of Education and Child Studies before data collection. It was also registered at the ISRCTN (registry number 11267699) after data collection. All participants gave informed consent. Data collection took place from May 2018 to January 2020.

### Study design

**Fig. 1:**
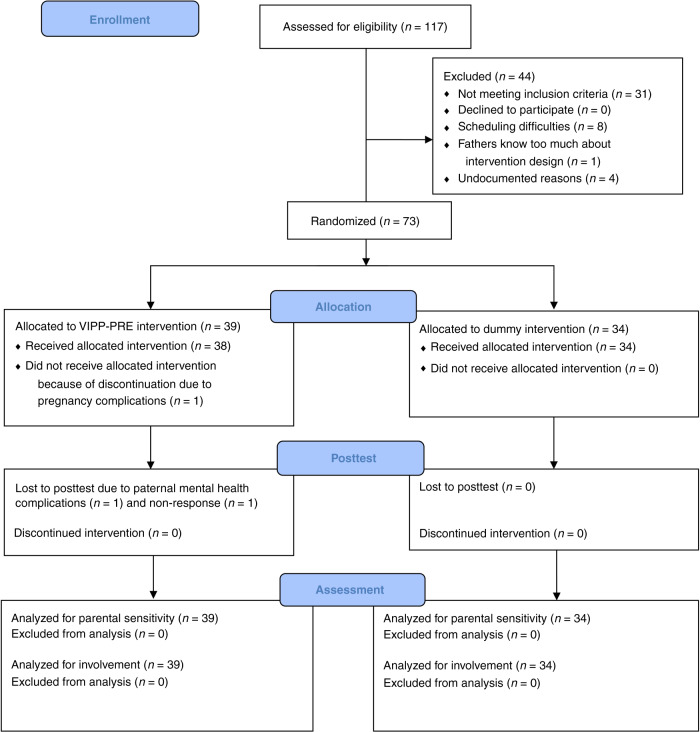
Inclusion. CONSORT flow diagram of enrollment, intervention allocation, postnatal posttest, and data analysis.

Figure 1 depicts a flow diagram of enrollment, intervention allocation, follow-up, and data analysis. The RCT included an experimental group (*n* = 39) and a control group (*n* = 34), to which participants were randomly assigned. Randomization was performed by one of the researchers before the start of the study using a computer-generated randomization sequence. Participants were enrolled by the second and third authors of this manuscript. The pretest and posttest visits were separate from the intervention sessions. At the end of the pretest visit, an envelope with the group assignment was opened and the participants were informed of their assignment. Attrition was absent in the control group and small in the experimental group (*n* = 2; we lost one participant after randomization due to pregnancy complications, and one after the intervention sessions due to mental health problems). Postnatal data on paternal sensitivity was missing for one participant in the experimental group. Postnatal data on paternal involvement was missing for seven participants in the experimental group and for three participants in the control group. Recruitment was stopped when we approached the end of the funding period. As recruitment was slower than expected (we used an opt-in model as requested by our ethics board, and the assessments, including neural imaging, required substantial time and dedication from participants), this resulted in a sample smaller than originally intended (*N* = 73 vs. the planned *N* = 140), and in somewhat unequal numbers in experimental and control groups. Using the software program G*Power, we computed what effects we could detect as significant with our sample. Power value was set at 0.80, alpha at 0.05, and correlation between pre- and posttest measures at 0.5, resulting in detectable effect sizes of Cohen’s *f* = 0.166 which corresponds to a Cohen’s *d* of 0.33. Pretest measures were administered at the gestational age of 20–32 weeks, followed by the intervention that usually started 1–2 weeks later. The posttest was administered approximately 9 weeks postnatally.

### Randomization

Randomization was not blind for participants or interveners, but the two arms of the study were presented to participants in similar ways, namely as an intervention focusing on interaction with their infants (VIPP-PRE), and an intervention discussing the pregnancy and the development of their unborn infants (phone calls). Researchers coding pre- and posttest data were blind to group membership.

### Intervention

#### VIPP-PRE

In VIPP-PRE, fathers are invited to interact with the fetus both verbally and by touching and softly massaging the infant through the mother’s abdominal wall in three sessions. The fetus is made visible through ultrasound. While recording, the intervener provides live feedback by using “speaking for the baby” techniques, and the father is stimulated to “read” his baby’s signals and behave accordingly. For example, the intervener may note: “she is quite active; she may hear your voice and she is at an age that she recognizes it” or “Look what happens now that you sung for him, he relaxes and sucks his thumb”. In addition, during the second and third sessions, after the live ultrasounds, the intervener presents the recordings of the previous session, pausing at relevant moments, and using a prepared script to comment on these fragments, following the themes of attachment and exploration (e.g., the difference between active, explorative behavior and a calm, resting posture as infant signals asking for different parental responses), speaking for the baby, and sensitivity chains.^7^ The first two sessions focus on building a working relationship and emphasize positive father–infant interactions. The third session also uses “corrective messages” to actively improve parenting behavior (i.e., discussing insensitive behavior and suggesting a more sensitive alternative).

During each ultrasound recording part of the session, the first minutes were shared with both parents, to accommodate mothers’ wishes to see their baby as well. However, the mothers were asked to stay aloof and read a magazine as soon as the interactions between the father and his unborn child started, and mothers were not present during the video-feedback part of the session. Upon request of the ethics committee, all sessions took place in a prenatal health clinic with an eye to incidental findings. The full intervention protocol is reported elsewhere.^7^ In the intervention group, one father missed two sessions; all other intervention fathers received three sessions. Twenty percent of the intervention scripts were reviewed in supervision sessions to ensure treatment fidelity.

#### Control condition

Fathers in the control group received phone calls from a researcher during the same period and with the same frequency as fathers receiving VIPP-PRE. Fathers were asked about the development of the pregnancy, any pregnancy-related activities or appointments, their preparations for birth and fatherhood, and communication about the pregnancy or the baby in their social network. In both groups, information on the current fetal developmental stage was provided during each session. In the control group, one father missed one session; all other control fathers received three sessions.

### Outcomes

#### Parental sensitivity

Parental sensitivity was assessed based on video-recorded observations both prenatally and postnatally. Prenatally, the infant simulator (RealCare Baby II-Plus; Realityworks, Eau Claire, WI) was used; a life-like doll resembling a young infant in appearance, size, and weight. Use of the infant stimulator has previously been shown to provide a reliable and valid way of assessing parental sensitivity^23,24^ and is also suitable for populations without children of their own. In a room with a PC with internet access as a competing activity and to increase the ecological validity of the situation (because in the home environment passive distractors are almost always present), participants were instructed to take care of the doll as if it were their own child, including careful handling such as offering neck support. They were told that the doll could cry just like a real infant. They could use various objects to soothe the child (i.e., a blanket, toys, bottle, diaper, and the second set of clothes), and could use the PC if they wanted to check their e-mail or do something else online. The simulator was programmed to be quiet for approximately 3 min, then cry for approximately 5 min, followed by being quiet for approximately 2 min. A similar protocol has been previously used.^25^ The cry sounds consisted of pre-set recordings of a real infant and increased in intensity during the 5-min cry episode. Unbeknownst to the participants, they were not able to effectively soothe the infant. During the postnatal posttest assessment, father–infant interactions were observed at home during a 10-min play session. Participants were instructed to engage in their usual routines of play, the first 5 min without play material and the last 5 min with play material. All videotaped father–doll and father–infant interactions were coded by independent coders using the Ainsworth Sensitivity scale^26^ with scores ranging from 1 (insensitive) to 9 (sensitive). Five coders were trained to code the father–doll interactions, and reliability with expert coders was adequate 0.69–0.79 (ICC, single measures, absolute agreement).^27^ Five different coders were trained to code the infant–father interactions (ICC = 0.68–0.76).

#### Involvement

Paternal involvement was prospectively assessed via an application on participants’ smartphones in the week after the pre- and post-tests. During this week, participants received six notifications per day to complete questions on their smartphones (at random times between 9 and 10 am, 11 and 12 am, 1 and 2 pm, 3 and 4 pm, 7 and 8 pm, and 9 and 10 pm). First, participants were asked whether they had thought about, spoken about, or communicated with their infant to measure cognitive/affective involvement.^28^ Second, accessibility (i.e., being present and available) was assessed by asking participants whether they were in the proximity of their partner (prenatal) or infant (postnatal) and—in case of postnatal assessments—if the infant was awake. When in the proximity, engagement (i.e., interacting with the infant) was measured by asking participants whether they had interacted (e.g., changed the infant’s diaper, felt the unborn infant kicking) or communicated with their (unborn) infant.

All responses were coded 0 = no, 1 = yes. In accordance with previous studies using a smartphone application for behavioral assessments,^29^ daily scores on cognitive/affective involvement and on accessibility were averaged for each participant over the 1-week period by dividing the sum of scores by the total number of responses. In addition, for each participant, a score for engagement relative to access to the infant or mother was calculated by dividing the sum of engagement across the one-week period by the sum of accessibility or (for postnatal assessments) the sum of times when the infant was awake across the 1-week period. Cognitive/affective involvement and engagement were correlated on pretest (*r* = 0.36, *p* = 0.003) and posttest (*r* = 0.33, *p* = 0.011). Since accessibility did not correlate with engagement at pre- and posttest (*r* < 0.05) and was correlated with cognitive/affective involvement only at posttest (*r* = 0.63, *p* < 0.001), cognitive/affective involvement and engagement were averaged into one component indicating involvement. Scores ranged from 0 to 1 with higher scores indicating more paternal involvement.

#### Covariates

Participants reported on the following demographic and health-related variables: age, average years of education following primary education, current depressive symptomatology, age of mother, infant gestational age at pretest, infant age at the posttest, infant sex, infant health, and pregnancy and birth complications.

### Data analysis

#### Preliminary analyses

Data inspection revealed that the continuous variables were normally distributed with no outlying values. Three percent (range: 0–11%) of the data was missing. Little’s MCAR test^30^ showed that values were missing completely at random (*χ*^2^ (24) = 34.60, *p* = 0.075). To follow an intent-to-treat approach, missing values were imputed by means of multiple imputation using the package “mice”^31^ in R.^32^ Betas were computed as estimates of effect sizes for continuous outcome variables.^33^

#### Main analyses

In line with the preregistration, two-level linear mixed effect models accounting for repeated measures of sensitivity and involvement over time (level 1) in individuals (level 2) were incrementally compared using the likelihood ratio test for imputed datasets with the “mitml” package.^34^ The final model included the fixed effects of time (coded as 0 = pretest, 1 = posttest), the main effect for condition (coded as 0 = Control, 1 = VIPP-PRE), and the interaction between time and condition. The significance of model and parameter estimates was determined at *α* = 0.05. In sensitivity analyses, models were tested in the non-imputed dataset and involvement analyses were repeated in participants with >33% valid responses on the posttest of the smartphone application. In addition, analyses on parental sensitivity were repeated including pretest scores on sensitivity as a covariate.

## Results

Figure 1 presents the number of participants at each time point. Characteristics of participants are summarized in Table 1. The intervention and control groups did not differ on potential covariates or pretest measures. Correlations between study variables are presented in Supplementary Table S1.

**Table 1:** Participant characteristics.

Measure	VIPP-PRE	Control	*t*/*χ*^2^^b^
Mean (SD)^a^	*n* = 39	*n* = 34	
Demographics			
Age father	32.57 (2.87)	32.66 (3.71)	−0.12
Education father	8.56 (1.48)	9.06 (1.37)	−1.47
Race father (% Caucasian)	93	97	−0.79
Age mother	30.05 (3.33)	30.95 (3.64)	−1.11
Gestational age infant at pretest (weeks)	24.91 (2.78)	25.00 (2.89)	−0.13
Age infant at posttest (weeks)	11.07 (4.77)	9.72 (2.41)	1.50
Infant sex (% boys)	38	38	0.00
Health-related variables			
Pregnancy complications (% yes)	35	56	−2.91
Birth complications (% yes)	41	47	−0.24
Infant health	−0.11 (0.70)	0.08 (0.79)	−1.02
Depressive symptoms father	5.03 (3.41)	5.12 (3.13)	−0.12
Outcome variables at pretest			
Sensitivity	5.34 (1.78)	6.11 (1.80)	−1.83
Involvement	0.42 (0.17)	0.40 (0.15)	0.42
Outcome variables at posttest			
Sensitivity	5.94 (1.56)	5.56 (1.57)	1.03
Involvement	0.81 (0.12)	0.76 (0.18)	1.42

Descriptives are calculated on the non-imputed data.

^a^Unless indicated otherwise.

^b^*χ*^2^ tests and *t*-test were performed to test whether there were pretest group differences between fathers in the VIPP-PRE and control conditions. No significant group differences were found.

### Sensitivity

**Fig. 2:**
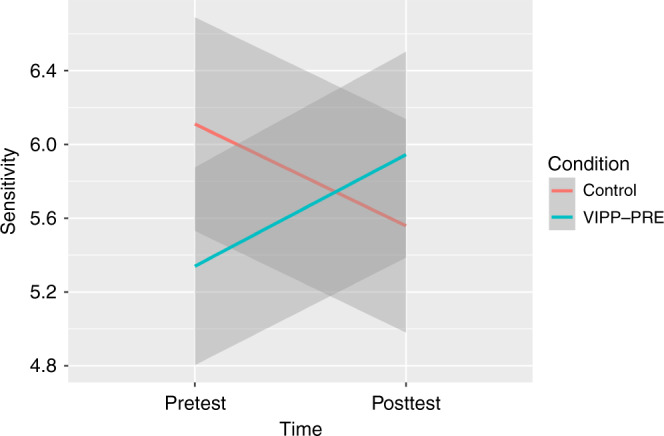
Paternal sensitivity before the birth (pretest) of their infant and after the birth of their infant (posttest). Data of complete cases are plotted. VIPP-PRE: Video-Feedback Intervention to Promote Positive Parenting Prenatal. Control: Dummy program consisting of telephone calls. The shadows surrounding the lines represent 95% confident interval levels.

The unconditional means model (Model 1) revealed that the proportion of explained variance (ICC) was 0.06 at the individual level. Adding the main effects of time and condition to the unconditional means model did not improve model fit (see Table 2). Adding the fixed effect of the interaction between time and condition significantly improved model fit, and the interaction effect was significant (*β* = 0.28, SE = 0.54, *p* = 0.043). A plot of this interaction effect (Fig. 2) revealed a decline in sensitivity in the control group (slope *β* = −0.16), and an increase in sensitivity in the intervention group (slope *β* = 0.16).

**Table 2:** Parameter estimates of the linear mixed model on sensitivity.

	Model 1	Model 2	Model 3
Fixed effects	Coef (se)	Coef (se)	Coef (se)
Intercept	5.71 (0.15)**	5.82 (0.25)**	6.11 (0.29)**
Time		0.03 (0.28)	−0.55 (0.39)
Condition		−0.23 (0.29)	−0.77 (0.39)*
Time × Condition			1.09 (0.54)*
Variance components (ICs)			
Individual level	0.16	0.15	0.22
Residual	2.71	2.71	2.56
Change in model fit (*F*)		0.33	3.96*

Unstandardized regression coefficients are displayed. Time: 0 = pretest, 1 = posttest; Condition: 0 = Control, 1 = VIPP-PRE.

*p < 0.05; **p < 0.01.

### Involvement

The unconditional means model (Model 1) revealed that the proportion of explained variance (ICC) was 0.00 at the individual level. Adding the fixed effects of time and condition significantly improved model fit (see Table 3). Time (*β* = 0.77, SE = 0.03, *p* < 0.001), but not condition (*β* = 0.07, SE = 0.03, *p* = 0.230), was significantly positively associated with involvement, meaning that fathers’ involvement increased over time regardless of their group status. Adding the interaction effect of time and condition did not improve model fit and the interaction was not significant (*β* = 0.05, SE = 0.05, *p* = 0.572).

**Table 3:** Parameter estimates of the linear mixed model on involvement.

	Model 1	Model 2	Model 3
Fixed effects	Coef (se)	Coef (se)	Coef (se)
Intercept	0.60 (0.02)**	0.40 (0.02)**	0.41(0.03)**
Time		0.37 (0.03)**	0.35 (0.04)**
Condition		0.03 (0.03)	0.02 (0.04)
Time × Condition			0.03 (0.05)
Variance components (ICs)			
Individual level	0.00	0.00	0.00
Residual	0.06	0.02	0.02
Change in model fit (*F*)		59.75**	0.34

Unstandardized regression coefficients are displayed. Time: 0 = pretest, 1 = posttest; Condition: 0 = Control, 1 = VIPP-PRE.

**p < 0.01.

### Sensitivity analyses

Analyses in the non-imputed dataset yielded similar outcomes as analyses on the multiply imputed datasets (see Supplementary Tables S2 and S3): the interaction of time × condition was significant for sensitivity (*p* = 0.033) and for involvement, only the main effect of time was significant (*p* < 0.001). Results of the mixed models on involvement in the sample with >33% valid answers on the posttest (*n* = 45) confirmed results in the total group, i.e., involvement increased from the prenatal to the postnatal phase (*p*s < 0.001). Finally, analysis of parental sensitivity including pretest scores on sensitivity as a covariate yielded similar outcomes: the interaction between condition and time remained significant (*β* = 0.28, *p* = 0.02).

## Discussion

This preregistered RCT (https://osf.io/487xc) of the first trial of VIPP-PRE demonstrated promising efficacy in improving sensitive fathering in the early postnatal phase. As hypothesized, fathers receiving the prenatal VIPP-PRE intervention showed a significant enhancement of their sensitive parenting across the perinatal period, relative to fathers receiving a dummy intervention with phone calls. Fathers’ involvement with their infants increased significantly from the prenatal to postnatal period, regardless of the intervention.

The positive effect of assisted interactions with the unborn child on fathers’ sensitive parenting aligns with considering the perinatal period as an important window of opportunity for intervening, with potential long-term positive effects for future father–child interactions.^14^ The transition to parenthood is often experienced as stressful for both mothers and fathers.^35^ However, compared to mothers, fathers may be neurobiologically and behaviorally less prepared for the birth of their child^19,36^ making their transition even more challenging. This may negatively affect their wellbeing and capacity to provide appropriate care for their infant.^37^ Yet, so far, only two other prenatal interventions that focus on parent–child interactions have included fathers.^17,18^ In line with our results, both interventions demonstrated a positive effect on father–child interaction quality. However, these interventions were either broad, impeding knowledge of effective components,^17^ or included many sessions.^18^ In contrast, the VIPP-PRE is a brief and focused intervention program promoting sensitive interaction between the father and his baby by using each specific dyad as its own model. Elsewhere we have developed a model of what we think are the active ingredients of the VIPP,^38^ which we further tested in a meta-analysis of the first 25 randomized controlled trials on the effects of the VIPP.^39^ For VIPP-PRE in particular, the opportunity to see their infant and see the infant responding to what happens seems to have an impact on the fathers. One father noted: “Seeing the baby and her reaction to my voice was special” and another father said: “I like having a moment to admire my son. To see that he responds to my presence and to follow his development better. These are precious moments to me”. Moreover, the interveners’ comments helped them to realize that the infant has a mind of their own and a behavioral status that makes them ready or not ready for interaction. Thus, over the sessions, interveners noted that fathers more carefully observed their baby on the ultrasound images before starting the interaction or verbalized their baby’s perspective.^40^ Our findings suggest that VIPP-PRE is a promising candidate for boosting the development of adequate parental care behaviors in the postnatal period when infants are highly receptive to the effects of caregiving quality.^37^ Whether such intervention effects remain observable over time is a question that has been meta-analytically answered in the affirmative for other versions of the VIPP program,^19^ but is an outstanding question for VIPP-PRE.

Our results indicate that involvement increased from the prenatal to the postnatal period, regardless of the intervention, in line with previous findings that fathers feel more responsible for their infants after birth.^41^ The physical presence of the infant and a growing bond may explain fathers’ increase in activities with their child. In our low-risk sample, these factors may be sufficient to elicit parental involvement. Moreover, the VIPP-PRE intervention focused less on enhancing paternal involvement than on paternal sensitivity to their infant’s signals and responding to these signals in prompt and adequate ways. The control condition may also have promoted involvement with the baby.

Our results should be cautiously interpreted as promising, given the limited power (see https://osf.io/487xc) of the modest sample size and the relatively homogeneous group of well-educated, motivated participants. It is tempting to suggest that results may be stronger rather than weaker in fathers who show initially low engagement with their partner’s pregnancy, but this hypothesis needs empirical evidence. Moreover, the effects of the intervention might be mediated through mothers or the relationship quality, which we did not examine in the current paper. The VIPP-PRE program may have helped to improve the partner and co-parenting relationship by providing the shared experience of extra ultrasounds, whereas in the control group only fathers and not mothers were involved in the phone calls. This may have led to differences between the two groups. However, the video-feedback was done without the mothers being present.^7^

Furthermore, pretest and posttest measures of sensitivity differed in that an infant doll was used at the pretest. Previous work has however shown adequate associations (*r* = 0.53) between parenting behavior with the doll and one’s own child.^24,42^ These correlations are equal to, or stronger than, stability of sensitivity in longitudinal studies starting after birth, strongly suggesting that we measured the same construct at different points in time. Lastly, the study design did not include a condition with ultrasounds without video-feedback, so the effects of VIPP-PRE cannot be distinguished from the effects of providing extra ultrasounds per se. Nevertheless, the effect size of VIPP-PRE (*d* = 0.49) is in line with the meta-analytic effect size on parental sensitivity of other VIPP trials (*d* = 0.47, 95% CI 0.34, 0.59, PI 0.24, 0.72).^19^ While the meta-analytic VIPP effects remained of similar strength over time,^19^ future research should examine whether this holds for VIPP-PRE as well.

In virtually all western countries, ultrasounds are part of the standard care during pregnancy. Ultrasound measures are currently used to monitor fetal growth and development but may also create an opportunity for stimulating father–infant interaction with feedback to promote postnatal caregiving quality. Of course, the current findings should be replicated in larger pragmatic randomized trials. A special focus may be on fathers who are difficult to reach and who might receive home visits with ultrasound imaging using the portable equipment that we used in the current study. The feasibility and acceptability of VIPP-PRE^7^ together with its efficacy as shown in the current preregistered RCT, suggest that prenatal video-feedback using ultrasound imaging of the child holds the potential to enhance the quality of parenting in an important but understudied group of parents, namely fathers, in a period that is critical in their lives and may have a lasting impact: the transition to fatherhood.

## Supplementary information

Supplementary Checklist

Supplementary Material

## Data Availability

The datasets generated during and/or analyzed during the current study are not publicly available to prevent compromising individual privacy but are available from the corresponding author on reasonable request.
