# Supplementary material for: Fathers’ sensitive parenting enhanced by prenatal video-feedback: a randomized controlled trial using ultrasound imaging
Source: Pediatr Res. 2022 Jul 29;93(4):1024–30. doi: 10.1038/s41390-022-02183-9 (PMC10033405; doi:10.1038/s41390-022-02183-9)
Supplement: Supplementary file 2 — Supplementary Material [file 41390_2022_2183_MOESM2_ESM.pdf]

**Table S1.** Pearson correlations separately for the control (in bold) and the intervention group

| | 1 | 2 | 3 | 4 |
|---|---|---|---|---|
| 1. Sensitivity pretest | - | **-.15** | **.47**** | **.01** |
| 2. Sensitivity posttest | .31 | - | **-.07** | **.29** |
| 3. Involvement pretest | -.17 | -.18 | - | **.10** |
| 4. Involvement posttest | -.06 | -.08 | .25 | - |

Note. Correlations for the control group are presented above the diagonal in bold and correlation for the intervention group are presented below the diagonal. * $p < .05$, ** $p < .01$.

**Table S2.** Parameter estimates of the linear mixed model on sensitivity with non-imputed data

| | Model 1 | Model 2 | Model 3 |
|---|---|---|---|
| Fixed effects | Coef (se) | Coef (se) | Coef (se) |
| Intercept | 5.73 (0.15)** | 5.81 (0.25)** | 6.11 (0.28)** |
| Time | | 0.05 (0.28) | -0.55 (0.39) |
| Condition | | -0.21 (0.29) | -0.77 (0.39)* |
| Time*Condition | | | 1.16 (0.53)* |
| Variance components (ICs) | | | |
| Individual level | 0.15 | 0.14 | 0.23 |
| Residual | 2.70 | 2.70 | 2.53 |
| Change in model fit ($X^2$) | | 0.54 | 4.55* |

*Note*. Unstandardized regression coefficients are displayed. Time: 0 = pretest, 1 = posttest; Condition: 0 = Control, 1 = VIPP-PRE. * $p < .05$, ** $p < .01$.

**Table S3.** Parameter estimates of the linear mixed model on sensitivity with non-imputed data

| | Model 1 | Model 2 | Model 3 |
|---|---|---|---|
| Fixed effects | Coef (se) | Coef (se) | Coef (se) |
| Intercept | 0.59 (0.02)** | 0.40 (0.02)** | 0.41 (0.03)** |
| Time | | 0.37 (0.02)** | 0.35 (0.04)** |
| Condition | | 0.03 (0.03) | 0.02 (0.04) |
| Time*Condition | | | 0.04 (0.05) |
| Variance components (ICs) | | | |
| Individual level | 0.00 | 0.00 | 0.00 |
| Residual | 0.06 | 0.02 | 0.02 |
| Change in model fit ($X^2$) | | 117.35** | 0.58 |

*Note*. Unstandardized regression coefficients are displayed. Time: 0 = pretest, 1 = posttest; Condition: 0 = Control, 1 = VIPP-PRE. * $p < .05$, ** $p < .01$.

In addition to the linear mixed effect models accounting for repeated measures over time (level 1) in individuals (level 2) with and without pretest sensitivity as a covariate, we performed a multiple regression analysis predicting posttest sensitivity from condition with pretest sensitivity entered in the first step. The results were in the same direction as in the main analysis, with overlapping confidence intervals of the effect size, but the effect of condition was not significant in the regression analysis, see Table S4.

**Table S4** Regression analysis predicting posttest sensitivity from condition with pretest sensitivity entered in the first step of the analysis

| | B | SE | *t* | *p* |
|---|---|---|---|---|
| Step 1 | | | | |
| Pretest Sensitivity | 0.13 | 0.11 | 1.22 | .23 |
| Step 2 | | | | |
| Condition | 0.45 | 0.38 | 1.18 | .24 |

*Note*. Unstandardized regression coefficients are displayed. Condition: 0 = Control, 1 = VIPP-PRE.
